# Postpartum Women’s Body Dissatisfaction: A Systematic Review of Theoretical Models and Regression Analyses

**DOI:** 10.3390/ijerph22091463

**Published:** 2025-09-22

**Authors:** Marcela Rodrigues de Siqueira, Tuany Mageste Limongi, Eduardo Borba Salzer, Ana Paula Delgado Bomtempo, Juliana Fernandes Filgueiras Meireles, Clara Mockdece Neves

**Affiliations:** 1 Graduate Program in Physical Education, Faculty of Physical Education and Sports, Federal University of Juiz de Fora, Juiz de Fora 36036-900, Brazil; marcela.rodrigues@estudante.ufjf.br (M.R.d.S.); eduardo.salzer@gmail.com (E.B.S.); ana.dbomtempo@gmail.com (A.P.D.B.); 2 Department of Physiotherapy, University of Vassouras, Vassouras 27700-000, Brazil; 3 Graduate Program in Health, Faculty of Medicine, Federal University of Juiz de Fora, Juiz de Fora 36036-900, Brazil; tuanymageste@gmail.com; 4 Department of Family and Community Medicine, School of Community Medicine, University of Oklahoma, Tulsa, OK 74120, USA; juliana-meireles@ouhsc.edu

**Keywords:** postpartum, body image, theoretical model, regression analysis, systematic review

## Abstract

Postpartum body image is a critical aspect of maternal well-being, influenced by sociocultural, psychological, and relational factors. Theoretical models offer a broader framework for understanding these influences, whereas regression analyses identify specific associations. This systematic review aimed to identify theoretical models assessing postpartum body image and to examine its association with relevant constructs using regression analysis. A search was conducted of four databases (PubMed, EMBASE, Web of Science, and American Psychological Association) between August 2022 and March 2024. Studies including mothers over 18 years old, within 0–24 months postpartum, that applied theoretical models and/or regression analyses were included. Of 169 articles retrieved from databases and 1 identified through backward snowballing, 18 studies met the inclusion criteria. Three theoretical models were identified, and highlighted sociocultural influences, social support, and breastfeeding as key determinants of body dissatisfaction. Regression analysis identified association between postpartum body image and maternal weight, depression, mode of delivery, sexual function, breastfeeding, and social support. Given these influences, the review emphasizes the importance of adopting holistic approaches to support maternal well-being. Interventions addressing postpartum body image should integrate strategies that consider cultural expectations, promote adequate social support, and address physical and emotional health challenges, such as weight management and mental health care. PROSPERO (CRD42022352992).

## 1. Introduction

The postpartum period is generally defined as the time following childbirth during which the maternal physiological and anatomical changes return to a non-pregnant state [1]. However, this timeframe varies across clinical and academic contexts. Some sources divide it into immediate, late, and remote phases [2,3], while others define it more narrowly, emphasizing the first 12 weeks after birth as a critical period for maternal care [4]. Several studies extend the postpartum period up to one year [2,5,6]. In the academic context, the term is sometimes applied in broader contexts, encompassing extended periods after childbirth [7]. Although it is recognized to begin immediately after childbirth, maternal recovery does not necessarily follow a linear course [2,3,5]. This process is influenced by physiological, hormonal, and psychosocial factors, underscoring the temporal imprecision of this stage [2,5,6,8]. It represents a critical moment for women, marked by significant physical and emotional changes, including body weight fluctuations, anxiety symptoms, concerns about infant care, feelings of sadness, and stress related to adapting to a new routine [9].

Women’s adaptation to these shifts has significant repercussions on their overall well-being and psychological health [10]. Psychological stressors inherent to motherhood, combined with heightened concerns about body image due to residual postpartum changes, can lead to significant emotional distress and an increased risk of eating disorders [11,12,13]. Body image is a complex construct conceptualized as the mental representation individuals have of their body, encompassing perceptions, thoughts, and feelings. Its complexity lies in the fact that it is shaped not only by internal factors such as emotions and cognition, but also by external psychological, social, and cultural influences that interact and vary across time and context [14,15]. The thin-body ideal as a sociocultural beauty standard can lead postpartum women to experience high levels of body dissatisfaction [16,17]. Negative evaluation of body size and shape are often due to increased pressure to achieve an unrealistic figure shortly after giving birth and the expectation to return to a pre-pregnancy state [15,16,17,18].

In recent years, the research interest in postpartum body image [19] has been increased. However, only a few systematic reviews [12,19,20,21,22,23] have specifically analyze the experiences of postpartum women, addressing body image in both quantitative and qualitative ways [12,20,21]. Evidence indicates that the transition to motherhood is often perceived as incompatible with other roles, such as those of wife, sexually attractive woman, or professional, with body dissatisfaction and efforts to regain the pre-pregnancy body being common [12,21]. Body dissatisfaction has been associated with prenatal and postpartum depression [20] and to an increased risk of depression and anxiety in the first year postpartum [22]. Greater body satisfaction, in turn, has been associated with higher likelihood of breastfeeding [23]. More recently, Lee et al. (2023) [19] conducted a systematic review using a socioecological framework, showing that postpartum body image is influenced by multiple interrelated factors, including mental health challenges, such as depression and anxiety, breastfeeding experiences and self-efficacy, and broader socioecological pressures related to cultural norms, media exposure, and partner expectations. These findings highlight the need for a comprehensive, multilevel approach to understanding and supporting postpartum body image.

Given the complexity of the construct, in-depth approach is essential to fully understand its multidimensional nature [10,14,15,24]. Theoretical models are valuable approach for studying and understanding body image as they offer a hypothetical framework that can be tested through obtained experiences and practices. This allows for a deeper understanding of the complex interaction between variables [25,26]. Regression based analysis, by using a pre-established theory, can specify the association between an outcome of interest and one or more variables, thus aiding in the explanation of the events in question [27,28]. These analyses are particularly valuable in understanding, predicting, and controlling how different variables are associated with body image [29,30,31,32].

Despite increasing interest in postpartum body image, there remains a lack of synthesis regarding how associated variables are explored through both theoretical frameworks and regression-based analyses. Previous systematic reviews have largely focused on descriptive or qualitative aspects, leaving a gap in understanding the statistical relationships and conceptual models underpinning this construct. Addressing this gap is essential to advance the field and support the development of effective interventions for postpartum women. This review therefore aims to identify and analyze existing theoretical models used to assess postpartum body image and to determine which variables have been statistically associated with this construct through regression-based analyses. Accordingly, this review is guided by the following research question: What theoretical models with diagrams have been used to assess postpartum body image, and which variables have been identified as significantly associated with this construct through regression-based analyses? For this review, regression-based analyses include both traditional regression methods and multivariate techniques such as path analysis and structural equation modeling (SEM), given their foundation in regression principles and their capacity to examine complex relationships among variables. By integrating these two approaches, the review provides a comprehensive perspective on methodological patterns, key influencing factors, and conceptual gaps—laying a foundation for future research and evidence-informed strategies to promote maternal well-being.

## 2. Materials and Methods

This review was conducted in accordance with the Preferred Reporting Items for Systematic Reviews and Meta-Analysis (PRISMA) [33] guidelines for reporting and was prospectively registered on PROSPERO (CRD42022352992).

### 2.1. Search Strategy

The search strategy was structured according to the PICO framework (Patient, Intervention, Comparison, and Outcome), tailoring subject headings for each database. Although the search covered studies from database inception to March 2024, the included studies were published between 2002 and 2023. Searches were conducted in the following databases: PubMed, EMBASE, Web of Science, and American Psychological Association (APA). All eligible primary studies published between database inception and March 2024 were included. For all databases, filters used in the search were studies on “humans”; articles in “Portuguese”, “English”, or “Spanish”. Additionally, studies identified through a backward snowball hand-search were also considered, meaning that the reference lists of the selected articles were examined to identify further relevant studies. The search terms combined related to the postpartum period (e.g., “postpartum”, “puerperium”), body image (e.g., “body dissatisfaction”, “body representation”), and statistical or theoretical modeling (e.g., “regression analysis”, “theoretical models”, “structural equation modeling”). The terms were adapted for each database. Full details of the search strategy are available in Appendix A.

### 2.2. Eligibility Criteria

Eligibility criteria applied were (a) studies with a population of women over 18 years of age; (b) postpartum period between 0 and 24 months [34,35]; (c) inclusion of body image components as a dependent and/or independent variable; and (d) studies that used theoretical model (with or without a diagrams) and/or regression analysis.

### 2.3. Data Collection and Analyses

Duplicate records were removed using Rayyan^®^ software. Two researchers (MRS and TML) independently and blindly reviewed the titles and abstracts of the citations retrieved by the search strategy, and the inclusion or exclusion decision was based on the eligibility criteria. The studies selected for full-text reading were compared and discussed, with agreement between the researchers regarding their inclusion or exclusion in this systematic review. A third reviewer (EBS) resolved any discrepancies. Data extraction was performed by one researcher (MRS) using a pilot form and verified by a second review (EBS) to ensure consistency. Extracted information included: authors, year of publication, country, sample size, age of the participants, postpartum period, instruments used, statistical analyses type performed, and main outcomes related to body image.

The studies were grouped according to the data analyses methods used to assess the content. A systematic narrative synthesis was used to present the review results in the form of text and table (see Table 1).

### 2.4. Risk of Bias Assessment

Risk of bias analyses were conducted by three independent researchers (MRS, TML, EBS) using standardized tools from the Joanna Briggs Institute (JBI). For cross-sectional observational studies, JBI Critical Appraisal Checklist for Analytical Cross-Sectional Studies (9 items) was applied, and for cohort studies, JBI Critical Appraisal Checklist for Cohort Studies (11 items) was used (see Table 2 and Table 3). Both tools share a similar structure, addressing methodological aspects such as sample adequacy, clarity of inclusion and exclusion criteria, validity and standardization of outcome measurements, identification and control of confounding factors, and consistency in analytical methods. Each item was rated as “yes,” “no,” “unclear,” or “not applicable,” allowing for a systematic and transparent appraisal of study quality. Cross-sectional studies were classified as high (7–9 “yes” responses), moderate (4–6), or low (1–3), while cohort studies were rated as high (8–11 “yes” responses), moderate (4–7), or low (0–3) [36,37,38].

## 3. Results

**Figure 1:**
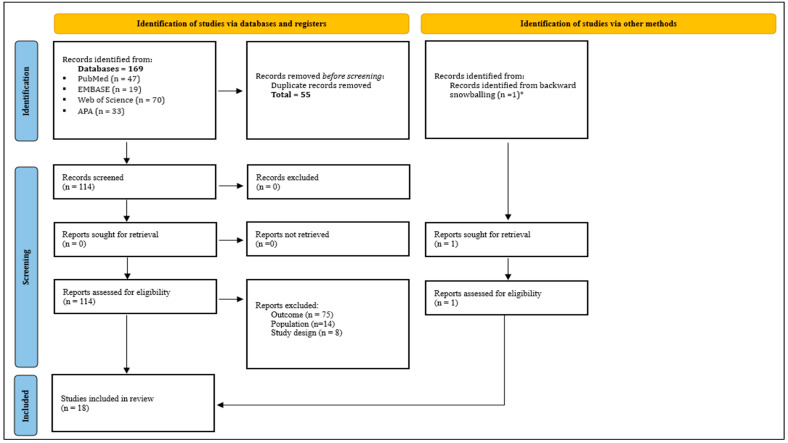
PRISMA flowchart. * Identified through a backward snowball hand-search [46].

The initial database search yielded 169 records, with one additional study identified in the backward snowball hand-search. In the screening process, a total 114 articles were assessed for eligibility. At the end of the entire process of screening, 18 studies were included. Figure 1 shows the PRISMA flowchart.

Most of the included studies (61.1%), were conducted in the United Stated of America [16,31,39,40,41,42,43,44,45,46,47], followed by Iran (11.1%) [18,48]. One study was identified from each of the following countries: China [49], Norway [34], Kenya [32], Turkey [29], and one study developed between Israel and the United Kingdom [50]. Regarding methodological strategies for data collection, the majority of the studies (88.8%) employed self-reported questionnaires, except for two studies [29,34] that used interviews responses.

The postpartum period in the studies ranged from immediate postpartum to 24 months, with a total sample of 44,360 women with sample sizes ranging from 61 to 39,915 participants, and an average age ranging from 21.9 to 34.4 years. Out of the total, 83.3% of the studies used regression analyses, while the remaining 16.6% developed theoretical models with diagrams [16,45,47]. The most common variables related to body image were weight, depression, age, type of delivery, sexual function, breastfeeding, social support, and sociocultural influences. The characteristics of the selected studies, the variables analyzed in relation to body image, the main instruments used, the statistical analyses, and the main outcomes are represented in Table 1.

**Table 1:** Characteristics of the included studies.

Author/Year Country	N Postpartum Period Age (Years)	Outcomes and Instruments Measured *	Type of Design Data Analysis	Outcomes Related to Body Image
Walker et al. (2002) [39] USA	N = 283 Postpartum period: immediately or less than 6 weeks Age: Anglo-American 22.8 (± 4.4), African American 22 (2 ± 3.8), Anglo-Hispanic 21.9 (± 3.4)	Body image—BCS Depression—CEDS	Descriptive correlational (cross-sectional) Hierarchical regression	Body image was correlated with depressive symptoms across all ethnicities. Weight was the main component related to body dissatisfaction.
Huang and Dai (2007) [49] China	N = 602 Postpartum period: less than 6 months Age: 30.22 (±4.35)	Lifestyle behavior and health promotion—Health Promoting Lifestyle Profile—Chinese version Depression—BDI Body image—BIS Social support perception—Interpersonal Support Evaluation List—Chinese brief version Health promotion behaviors—Self-Rated Abilities for Health Practices Scale—Chinese version	Descriptive correlational (cross-sectional) Multiple linear regression	The greater the weight retention during the postpartum, the lower the perceived satisfaction with body image.
Downs et al. (2008) [40] USA	N = 230 Postpartum period: less than 6 weeks Age: 30.05 (±4.13)	Depression—CEDS Body image—BSS Physical activity on leisure time—Leisure-Time Exercise Questionnaire	Prospective longitudinal cohort study Hierarchical regression	Body image satisfaction was negatively correlated with depressive symptoms.
Sweeney and Fingerhut (2013) [41] USA	N = 46 Postpartum period: less than 2 months Age: 27.17 (±6.59)	Depression—Postpartum Depression Predictors Inventory–Revised Body image—BAQ Maladaptive perfectionism—combination of three subscales: Almost Perfect Scale-Revised; Concern Over Mistakes subscale Doubts About Actions subscale of Frost’s Multidimensional Perfectionism Scale Depression—EPDS	Prospective longitudinal cohort study Hierarchical multiple regression	Third-trimester of pregnancy body dissatisfaction was identified as a risk factor for postpartum depression.
Walker et al. (2013) [42] USA	N = 419 Postpartum period: less than 6 weeks Age: 22.3 (±3.9)	BMI Depression—CEDS Body image—BCS Health behaviors—Self-Care Inventory	Descriptive correlational (cross-sectional) Poisson and multiple linear regression	Higher BMI was associated with increased body dissatisfaction.
Han et al. (2016) [34] Norway	N = 39.915 Postpartum period: less than 18 months Age: NR	BMI Depression—Hopkins Symptom Checklist—Short version Body image—Created by the authors	Prospective longitudinal cohort study Binary logistic regression	Concerns about body image mediated the association between increasing weight and the depressive symptoms, regardless of weight.
Walker et al. (2016) [43] USA	N = 168 Postpartum period: less than 6 months Age: Lower income group: 28.9 (±5.6) Higher income group: 32.8 (±4.8)	Stress—Perceived Stress Scale—4-item version Social support—Created by the authors Health behaviors—Diet, physical activity, smoking, and alcohol-use habits accessed by the authors in a previous study. Depression—CEDS Body image—Created by the authors	Descriptive correlational (cross-sectional) Linear and logistic regression	Greater social support was correlated with lower body dissatisfaction.
Jawed-Wessel et al. (2017) [44] USA	N = 168 Postpartum period: less than 12 months Age: 29.72 (±4.03)	Sexual function—Sexual Function Questionnaire Body image—BSS and Body-Image Self-Consciousness Scale Genital self-image—FGSIS	Descriptive correlational (cross-sectional) Hierarchical multiple regression	Body satisfaction/dissatisfaction varied according to the type of delivery. Negative correlation: body dissatisfaction with body awareness during intimacy increased. Body dissatisfaction with genital self-image and age. Sociocultural pressure from media, partners, and friends significantly predicted body dissatisfaction mediated by the thin ideal.
Lovering et al. (2018) [16] USA	N = 474 Postpartum period: less than 6 months Age: 30.9 (±4.5)	Body image influence—Subscales of Sociocultural Attitudes Towards Appearance Questionnaire-3, Perceived Sociocultural Pressure Scale, Family Influence Scale, Media Influence Scale, Peer Influence Scale, Partner Influence Scale Preocupation—Perceived preoccupation related to weight and diet, Perceived Family/Friend/Partner Preoccupation with Weight and Dieting Scale, Physical Appearance Comparison—Physical Appearance Comparison Scale Body image—Body Dissatisfaction subscale of EDI Bulimia—Bulimia subscale of EDI Body image muscularity- 10-item Muscularity for Women Scale Self-Esteem—RSES Depression—CEDS	Descriptive correlational (cross-sectional) Structural equation modeling	Sociocultural pressure from media, partners, and friends significantly predicted body dissatisfaction mediated by the thin ideal.
Rodgers et al. (2018) [45] USA	N = 151 Postpartum period: less than 6 months Age: 32.77 (±4.47)	Body Surveillance—Surveillance subscale of the Objectified Body Consciousness Body image—Body Dissatisfaction subscale of EDI Eating disorder—Eating Disorders Diagnostic Scale Depression—Depression, Anxiety and Stress Scale 21-item Short Form Subscale Breastfeeding—BSES Short-Form Breastfeeding barriers—Created by the authors Weight loss desire- To assess for desired weight loss, participants were asked to report current weight and post-pregnancy goal weight	Descriptive correlational (cross-sectional) Path analysis	Positive correlation: Body dissatisfaction with desired weight loss, body surveillance, breastfeeding barriers, depressive symptoms, and eating disorders. Negative correlation: Body dissatisfaction with breastfeeding self-efficacy.
Shloim et al. (2019) [50] Israel and United Kingdom	N = 61 Postpartum period: less than 24 months Age: Israel: 30.4 (±3.8) United Kingdom: 34.4 (±3.2)	BMI Self-Esteem—RSES Body image—BIS, Stunkard figure rating scale and Body Image Disturbance Questionnaire Eating behavior—Dutch Eating Behaviour Questionnaire	Prospective longitudinal cohort study Hierarchical linear modeling	Body image dissatisfaction had a positive correlation with BMI.
Schlaff et al. (2020) [46] USA	N = 269 Postpartum period: less than 12 months Age: 30.4 (±3.9)	Physical activity—Self-reported moderate to vigorous physical activity Body image—BAQ Depression—CEDS	Descriptive correlational (cross-sectional) Multiple linear regression	Postpartum body satisfaction was negatively correlated with postpartum depressive symptoms.
Tavakoli et al. (2021) [18] Iran	N = 300 Postpartum period: less than 6 months Age: 29.77 (±5.9)	BMI Fertility—Fertility Questionnaire Body image—Body Shape Questionnaire 34-item—Persian Version	Descriptive correlational (cross-sectional) Multiple linear regression	BMI was the main predictor to body dissatisfaction.
Kapa et al. (2022) [31] USA	N = 204 Postpartum period: between 2 weeks and 6 months Age: Between 27 and 30 years old.	Breastfeeding—Maternal Breastfeeding Evaluation Scale Breastfeeding—BSES –Short Form Eating disorder—Eating Attitudes Test—26 Body image—MBSRQ	Descriptive correlational (cross-sectional) Multiple linear regression	Body satisfaction was positively correlated with breastfeeding self-efficacy and experiences.
Kariuki et al. (2022) [32] Kenya	N = 567 Postpartum period: less than 10 weeks Age: 25.9	Depression—BDI Body image—Created by the authors	Descriptive correlational (cross-sectional) Multiple linear regression	Body dissatisfaction was positively correlated with depression
Rodgers et al. (2022) [47] USA	N = 156 Postpartum period: less than 6 weeks Age: 32.7 (±4.7)	Body image influence—Perceived Sociocultural Pressure Scale Partner support—Postpartum Partner Support Scale Thin Ideal—Subscale of the Sociocultural Attitudes Toward Appearance Questionnaire Depression- Depression, Anxiety, and Stress Scale-7 Body image—Body Dissatisfaction subscale of EDI Breastfeeding—BSES Eating disorder—Eating Disorder Diagnostic Scale	Descriptive correlational (cross-sectional) Path analysis	Body dissatisfaction was positively correlated with high levels of internalization of the thin ideals and partner appearance pressure.
Bakhteh et al. (2023) [48] Iran	N = 150 Postpartum period: less than 6 weeks Age: 28.60 (±4.5)	Compassion—Short Form Self-Compassion Questionnaire Body image—MBSRQ Depression—EPDS	Descriptive correlational (cross-sectional) Linear regression	Negative body image was a predictor of postpartum depression symptoms.
Acar Bektaş and Öcalan (2023) [29] Turkish	N = 197 Postpartum period: less than 1 week Age: 29.33 (±5.88)	Self-Esteem—RSES Body image—Body-Esteem Scale Genital self-image—FGSIS	Descriptive correlational (cross-sectional) Multiple linear regression	There was a significant and positive relationship between women’s body image and their genital self-image.

Note: Variable values are expressed exactly as described in each study. Abbreviations: BCS: Body Cathexis Scale; CEDS: Center for Epidemiologic Studies Depression Scale; BIS: Body Image Scale; BDI: Beck’s Depression Inventory; BSS: Body Satisfaction Scale; BAQ: Body Attitudes Questionnaire; EPDS: Edinburgh Postpartum Depression Scale; MBSRQ: Multidimensional Self-Body Relationships Questionnaire; RSES: Rosenberg Self-Esteem Scale; FGSIS: Female Genital Self-Image Scale; BSES: Breastfeeding Self-Efficacy Scale; EDI: Eating Disorder Inventory; BMI: body mass index.

The relationships between postpartum body image and other variables were examined in three studies that developed theoretical models with visual diagrams [16,45,47]. One model was an adaptation of the Tripartite Influence Model [16], while the other two focused on developing a biopsychosocial model [45,47]. The Tripartite Influence Model, based on sociocultural theory, posits those sociocultural pressures (parents, friends, media) mediated by social comparison and internalization of appearance ideals, predict body image-related outcomes [51]. Lovering et al. [16] adapted the original model for postpartum women, incorporating partners as a sociocultural influence. The adapted model by Lovering et al. [16] revealed that sociocultural pressures from media, partners, and friends significantly predicted body dissatisfaction through the mediating of the thin ideal, with media influence emerging as the strongest predictor [16].

Rodgers et al. (2018) [45] proposed the biopsychosocial model of body image and eating attitudes for postpartum women up to six months. The model included psychological factors, analyzed through depressive symptoms, and biological factors related to concerns about body weight. As a sociocultural variable, the model highlights body surveillance through self-objectification [45]. Within this framework, body dissatisfaction emerged as the outcome of desired weight loss, body surveillance, depressive symptoms, and breastfeeding barriers. In turn, body dissatisfaction was a predictor of lower breastfeeding self-efficacy and an increased risk of eating disorders.

The third study with a theoretical model aimed to expand the biopsychosocial model of body image, eating concerns, and breastfeeding, including the partner. Greater postpartum partner support significantly predicted greater breastfeeding self-efficacy, lower depressive symptoms, and less body dissatisfaction. Conversely, greater internalization of the thin ideal predicted higher levels of depression and body dissatisfaction, and lower breastfeeding self-efficacy. Additionally, greater partner pressure related to appearance was significant predictor of greater body dissatisfaction [47].

Fifteen articles [18,29,32,34,39,40,41,42,44,46,48,49,50] assessed the relationship between variables using regression analysis. The findings showed that variables associated with body image included: weight and body mass index (BMI), depressive symptoms, age, mode of delivery, sexual functioning, breastfeeding, and social support.

Fifteen articles [18,29,32,34,39,40,41,42,44,46,48,49,50] assessed the relationship between variables using regression analysis. The findings showed that variables associated with body image using regression analysis included: maternal weight and body mass index (BMI), depressive symptoms, age, type of delivery, sexual functioning, breastfeeding, and social support.

Two studies analyzed the association between postpartum body image, age, and mode of delivery, as well as their relationship with sexual function [29,44]. The findings of Jawed-Wessel et al. (2017) indicated that older maternal age and cesarean delivery were predictors of greater body dissatisfaction [29,44], and that women with a more positive genital self-image tended to report lower body dissatisfaction, which could significantly influence sexual function [29,44]. One study examined the relationship between self-efficacy, breastfeeding experiences, and body image [31], demonstrating that higher breastfeeding self-efficacy and more positive breastfeeding experiences were predictors of lower body dissatisfaction. Finally, one study explored the association between social support and body image, finding that greater social support was a significant predictor of lower body dissatisfaction [43].

Most studies employed descriptive correlational (cross-sectional) designs [16,18,29,31,32,39,42,43,44,45,46,47,48,49], while the remaining studies used prospective longitudinal cohort designs [34,40,41,50].

The risk of bias assessment for the 18 included studies is summarized in Table 2 and Table 3. Among the cross-sectional studies, the majority were rated as having moderate risk of bias (*n* = 9; 69%), with most employing descriptive correlational designs [29,31,39,42,43,44,45,46,47,48,49]. Only one cross-sectional study was rated as having low risk of bias [49]. Regarding the cohort studies, half were rated as having low risk of bias [34,40], while the remaining two were classified as moderate risk of bias [41,50].

**Table 2:** Risk of bias analysis of the included studies—JBI Critical appraisal checklist for analytical cross-sectional studies.

Author/Year	Q1	Q2	Q3	Q4	Q5	Q6	Q7	Q8	Q9	Total Score
Walker et al. (2002) [39]	Y	N	N	Y	Y	Y	U	Y	N	Moderate
Huang and Dai (2007) [49]	Y	Y	Y	Y	U	Y	U	Y	Y	High
Walker et al. (2013) [42]	Y	N	N	Y	N	Y	N	Y	N	Moderate
Walker et al. (2016) [43]	Y	N	N	Y	N	Y	Y	Y	Y	Moderate
Jawed-Wessel et al. (2017) [44]	Y	N	N	Y	Y	Y	U	Y	U	Moderate
Lovering et al. (2018) [16]	U	N	N	Y	U	Y	U	Y	U	Low
Rodgers et al. (2018) [45]	U	N	N	Y	U	Y	U	Y	U	Low
Schlaff et al. (2020) [46]	Y	N	N	Y	N	Y	U	Y	N	Moderate
Tavakoli et al. (2021) [18]	Y	Y	Y	Y	U	Y	N	Y	Y	Low
Kapa et al. (2022) [31]	Y	N	N	Y	N	Y	U	Y	Y	Moderate
Kariuki et al. (2022) [32]	Y	Y	Y	Y	Y	Y	U	Y	Y	Low
Rodgers et al. (2022) [47]	Y	N	N	Y	U	Y	N	Y	N	Moderate
Bakhteh et al. (2023) [48]	Y	Y	Y	N	Y	Y	U	Y	U	Moderate
Acar Bektaş and Öcalan (2023) [29]	N	Y	Y	N	Y	Y	U	Y	N	Moderate

Q1. Was the sample frame appropriate to address the target population? Q2. Were study participants sampled in an appropriate way? Q3. Was the sample size adequate? Q4. Were the study subjects and the setting described in detail? Q5. Was the data analysis conducted with sufficient coverage of the identified sample? Q6. Were valid methods used for the identification of the condition? Q7. Was the condition measured in a standard, reliable way for all participants? Q8. Was there appropriate statistical analysis? Q9. Was the response rate adequate, and if not, was the low response rate managed appropriately? Legend: Y = yes, N = no, U = unclear. Total score: high = 7–9 Y, moderate = 4–6 Y, low = 1–3 Y.

**Table 3:** Risk of bias analysis of the included studies—JBI Critical appraisal checklist for cohort studies.

Author/Year	Q1	Q2	Q3	Q4	Q5	Q6	Q7	Q8	Q9	Q10	Q11	Total Score
Downs et al. (2008) [40]	Y	Y	U	Y	U	U	Y	Y	U	U	Y	Moderate
Sweeney and Fingerhut (2013) [41]	Y	Y	U	Y	U	U	Y	Y	U	U	Y	Moderate
Han et al. (2016) [34]	Y	Y	U	Y	U	Y	U	Y	U	U	Y	Moderate
Shloim et al. (2019) [50]	Y	Y	U	U	U	Y	Y	Y	U	U	Y	Moderate

Q1. Were the two groups similar and recruited from the same population? Q2. Were the exposures measured similarly to assign people to both exposed and unexposed groups? Q3. Was the exposure measured in a valid and reliable way? Q4. Were confounding factors identified? Q5. Were strategies to deal with confounding factors stated? Q6. Were the groups/participants free of the outcome at the start of the study (or at the moment of exposure)? Q7. Were the outcomes measured in a valid and reliable way? Q8. Was the follow up time reported and sufficient to be long enough for outcomes to occur? Q9. Was follow up complete, and if not, were the reasons to loss to follow up described and explored? Q10. Were strategies to address incomplete follow up utilized? Q11. Was appropriate statistical analysis used? Legend: Y = yes, N = no, U = unclear. Total score: moderate = 4–7 Y.

## 4. Discussion

The aim of this review was to identify and analyze available theoretical models with diagrams to assess body image in postpartum women and understand the relationship between body image and the main constructs associated with it through regression models. This was done to identify which variables may have a significant impact on body image perception during the postpartum period.

This review identified three studies [16,45,47] that developed theoretical models to explain the relationships between body image and other variables. The model proposed by Lovering et al. [16] suggests that postpartum women experience sociocultural pressures to achieve unrealistic body shapes/sizes. The biopsychosocial model [45] demonstrated that self-surveillance contributed to body dissatisfaction, increasing barriers to breastfeeding. The expanded version of biopsychosocial model [47] highlights the influence of the partner, where greater appearance-related pressure reported by the partner was significantly correlated with greater body dissatisfaction. A common aspect among the analyses was the evaluation of breastfeeding and social support variables. Two of the three studies included partner support [16,47], while two assessed breastfeeding [45,47], highlighting key aspects of the postpartum period. These findings highlight the importance of considering not only individual variables but also social and relational factors when analyzing postpartum body image.

These models reveal a complex dynamic that can significantly impact the postpartum experience for women. The pressure to conform to unrealistic body standards, amplified by the media and social networks, intensifies concerns about body image, compromising both the psychological well-being and eating behaviors of new mothers [16]. Heightened self-surveillance to meet these sociocultural expectations not only increases body dissatisfaction, but also creates barriers to breastfeeding, a vital practice for maternal and infant health [45]. While partner support can alleviate body dissatisfaction, reduce the risk of depression, and enhance the likelihood of breastfeeding, any pressure from the partner regarding appearance can exacerbate these insecurities [47]. The interplay of these factors underscores the importance of valuing the body’s functions, reducing sociocultural pressures, and fostering a positive role for partners in promoting the physical and mental well-being of postpartum women [16].

Theoretical frameworks are fundamental for advancing the understanding of body image in the postpartum period, as they enable a more in-deep exploration of the associations among variables [45,47]. Although the Tripartite Influence Model was not directly applied in any of the included studies, its core principles underpinned the conceptual adaptation proposed by Lovering et al. (2018) [16], reflecting its foundational role in body image research. Within the studies reviewed, the sociocultural framework [16] was evaluated using structural equation modeling (SEM), whereas the biopsychosocial frameworks [45,47] applied path analysis. Although both approaches were grounded in cross-sectional designs, path analysis can offer insights into potential causal pathways, while SEM supports causal inference and the testing and validation of models involving intricately interrelated variables [16,26]. The findings of this review emphasize the need for more rigorous methodological strategies that integrate conceptual models with advanced statistical techniques to deepen the understanding of the complex set of factors influencing women during this period.

Variables related to body image in postpartum women were identified through studies using regression techniques. While these techniques have distinct characteristics and specific applications, they all specify the association between an outcome and one or more variables based on a pre-established theory [27,28]. Employing this type of analysis, most studies reported outcomes related to maternal weight and depressive symptoms, while a smaller number investigated age, mode of delivery, and sexual function [18,29,32,34,39,40,41,42,44,46,48,49,50].

Key points of convergence emerged between regression analyses and the reviewed theoretical models. Body mass index (BMI) was identified as an important predictor of body dissatisfaction in postpartum women, appearing both in the postpartum-adapted model [16] and in several regression studies [18,34,39,42,49,50]. Findings by Huang and Dai (2007) [49] also indicated that body image predicts postpartum weight retention, with higher levels of retained weight associated with greater body dissatisfaction. Although the biopsychosocial model [45] did not directly include BMI, it identified the desire for weight loss as a predictor of body dissatisfaction.

Depressive symptoms represent another common finding. Most regression analyses indicated that body dissatisfaction predicts depressive symptoms [32,40,45,48]. Longitudinal studies [34,41] suggest that body dissatisfaction may act as a risk factor for the development of depressive symptoms, particularly during the postpartum period. In the biopsychosocial model [45], depressive symptoms were direct predictors of body dissatisfaction. Furthermore, depressive symptoms are important indicators of maternal emotional well-being and, when neglected, can compromise caregiving practices and mother–infant interactions [39,42]. Therefore, assessing body image and depressive symptoms during pregnancy and the postpartum period is crucial, as a positive body image may serve as a protective factor against the development of depression [34].

Other outcomes analyzed include maternal social support and breastfeeding. Walker et al. (2016) [43] demonstrated that social support is associated with lower body dissatisfaction. In the expanded biopsychosocial model [47], greater partner support during the postpartum period was significantly associated with lower body dissatisfaction and higher breastfeeding self-efficacy. Complementarily, Kapa et al. (2022) [31] showed that higher breastfeeding self-efficacy and more positive maternal experiences during this period were associated with lower body dissatisfaction.

In the original biopsychosocial model [45], body dissatisfaction predicted lower breastfeeding self-efficacy. Considering that the postpartum period involves adaptation and acceptance of bodily changes, perceived social support—from partners, family, or friends—may promote a more positive evaluation of appearance and overall body health [52], fostering greater attention to bodily functions, such as breastfeeding, and less focus on physical appearance [31].

The studies included in this systematic review did not justify the temporal cutoff used to define the postpartum period. However, the literature applies different temporal boundaries, such as up to 12 months postpartum, or broader and less precise periods often referred to as remote postpartum, which may vary according to the duration of lactation and the return of menstruation [2]. These considerations highlight the need for greater clarity in defining the postpartum period, and it is important that future studies explicitly report this delimitation.

Most of the included studies were rated as having a moderate risk of bias, while one was classified as low risk and four as high risk. Common sources of bias included unclear reporting of sample size adequacy, insufficient detail in data analysis coverage, and standardization of condition measurement. These limitations affect the overall strength and reliability of the findings, particularly when interpreting associations identified in regression models. While the consistency of certain variables across studies adds weight to the conclusions, the moderate to high risk of bias in several cases underscores the need for cautious interpretation and highlights the importance of improving study design and reporting in future research.

While this systematic review provides valuable insights into the relationship between postpartum body image and associated factors, some limitations warrant consideration. First, most studies did not provide adequate details on the sampling method, raising concerns about the representativeness of the participants and limiting the interpretation and generalization of the results. Second, a disproportionate sample size contributed by a single study, which accounted for the majority of participants included in this review, potentially introducing bias in the overall findings. Third, the variety of instruments used to assess body image and other constructs, including author-developed questionnaires, represents a limitation. Moreover, the lack of detailed sociodemographic information made it difficult to characterize the respondents’ profiles. Another limitation is that our analysis could not account for the impact of different sociocultural norms on body image, as the included studies were conducted across diverse countries. Furthermore, most studies were cross-sectional, which restricts the ability to assess changes in body image over time and to capture how women’s expectations and experiences may shift across different stages of the postpartum period. This variability can influence how body image is perceived and reported and should be considered when interpreting the findings. Since the included studies were conducted in different cultural contexts, it is also important to recognize that body image expectations are shaped by sociocultural norms, which may differ widely and influence how women perceive and relate to their bodies during the postpartum period.

The findings of this study underscore the complexity of postpartum body image and the need for approaches that integrate individual, social, and cultural factors. Theoretical models provide a comprehensive framework for understanding these multiple influences, whereas regression analyses identify specific associations among relevant variables, particularly maternal weight (BMI), depressive symptoms, breastfeeding experiences, and social support. These results highlight the importance of early assessment of body dissatisfaction and interventions that promote realistic expectations regarding bodily changes, strengthen maternal self-efficacy, and encourage social support throughout the postpartum period. For clinical practice, these findings emphasize the value of integrated care strategies that include mental health screening and culturally sensitive guidance related to body image and breastfeeding. They may also help patients normalize their experiences, reduce stigma, and foster self-compassion. Finally, future research should use longitudinal designs to further explore these relationships and examine how factors such as culture, socioeconomic status, and partner dynamics shape body image over time.

## 5. Conclusions

This systematic review highlights the complex interaction between postpartum body image and various factors, including sociocultural pressures, psychological variables, and relational dynamics. Theoretical models emphasize the role of media influence, self-surveillance, and partner support in shaping body dissatisfaction, while regression analyses reveal consistent associations with maternal weight, depressive symptoms, mode of delivery, and breastfeeding experiences. Several of these findings, such as the impact of BMI and perceived social support, align with constructs present in the identified theoretical frameworks. However, other variables, like breastfeeding experiences and maternal self-efficacy, are less frequently represented in traditional models. This underscores the need to expand or adapt existing theories to better account for the postpartum context and to guide future research and intervention strategies.

## Data Availability

No new data were created or analyzed in this study. Data sharing is not applicable to this article.

## Abbreviations

The following abbreviations are used in this manuscript:

PRISMA	Preferred Reporting Items for Systematic Reviews and Meta-Analysis
PICO	Patient, Intervention, Comparison, and Outcome
APA	American Psychological Association
BCS	Body Cathexis Scale
CEDS	Center for Epidemiologic Studies Depression Scale
BIS	Body Image Scale
BDI	Beck’s Depression Inventory
BSS	Body Satisfaction Scale
BAQ	Body Attitudes Questionnaire
EPDS	Edinburgh Postpartum Depression Inventory
MBSRQ	Multidimensional Self-Body Relationships
RSES	Rosenberg Self-Esteem Scale
FGSIS	Female Genital Self-Image Scale
BSES	Breastfeeding Self-Efficacy Scale
EDI	Eating Disorder Inventory
BMI	body mass index
NR	Not reported
USA	United States of America
SEM	Structural Equation Modeling

## Appendix A

Search strategy—PUBMED

1. ((Postpartum Period) OR (Period, Postpartum)) OR (Postpartum)) OR (Postpartum Women)) OR (Women, Postpartum)) OR (Puerperium)
2. (((((((((((((((((((((((((((((((((((((((((((Latent Class Analysis) OR (Latent Class Analysis)) OR (LatentClass Analyses)) OR (Latent Variable Modeling)) OR (Latent Variable Modelings)) OR (Modeling, Latent Variable)) OR (Variable Modeling, Latent)) OR (Structural Equation Modeling)) OR (Modeling, Structural Equation)) OR (Structural Equation Modelings)) OR (Probabilistic Latent SemanticAnalysis)) OR (Latent Class Model)) OR (Latent Class Models)) OR (Model, Latent Class)) OR (Models, Theoretical)) OR (Models, Theoretical)) OR (Model, Theoretical)) OR (Theoretical Model)) OR (Theoretical Models)) OR (Models, Theoretic)) OR (Models (Theoretical))) OR (Experimental Model)) OR (Model, Experimental)) OR (Models, Experimental)) OR (Experimental Models)) OR (Mathematical Model)) OR (Mathematical Models)) OR (Model, Mathematical)) OR (Models, Mathematical)) OR (Theoretical Study)) OR (Studies, Theoretical)) OR (Study, Theoretical)) OR (Theoretical Studies)) OR (Regression Analysis)) OR (Regression Analysis)) OR (Analysis, Regression)) OR (Analyses, Regression)) OR (Regression Analyses)) OR (Regression Diagnostics)) OR (Diagnostics, Regression)) OR (Statistical Regression)) OR (Regression, Statistical)) OR (Regressions, Statistical)) OR (Statistical Regression)
3. ((((((((((((((((((((((Body Images) OR (Image, Body)) OR (Body Identity)) OR (Identity, Body)) OR (Body Representation)) OR (Body Representations)) OR (Representation, Body)) OR (Body Schema)) OR (Body Schemas)) OR (Schema, Body)) OR (Dissatisfaction, Body)) OR (Body ImageDissatisfaction)) OR (Body Image Dissatisfactions)) OR (Dissatisfaction, Body Image)) OR (Dissatisfactions, Body Image)) OR (Image Dissatisfaction, Body)) OR (Image Dissatisfactions, Body)) OR (Negative Body Image)) OR (Body Image, Negative)) OR (Body Images, Negative)) OR (Image, Negative Body)) OR (Images, Negative Body)) OR (Negative Body Image)
4. 1 AND 2 AND 3

Search strategy—WEB OF SCIENCE

ALL = ((((((Postpartum Period) OR (Period, Postpartum)) OR (Postpartum)) OR (Postpartum Women)) OR (Women, Postpartum)) OR (Puerperium))

1. (((((((((((((((((((((((((ALL = (Latent Class Analysis)) OR ALL = (Latent Class Analysis)) OR ALL = (Latent Class Analyses)) OR ALL = (Latent Variable Modeling)) OR ALL = (Latent Variable modeling)) OR ALL = (Modeling, Latent Variable)) OR ALL = (Variable Modeling, Latent)) OR ALL = (Structural Equation Modeling)) OR ALL = (Modeling, Structural Equation)) OR ALL = (Structural Equation modeling)) OR ALL = (Probabilistic Latent Semantic Analysis)) OR ALL = (Latent Class Model)) OR ALL = (Latent Class Models)) OR ALL = (Model, Latent Class)) OR ALL = (Models, Theoretical)) OR ALL = (Models, Theoretical)) OR ALL = (Model, Theoretical)) OR ALL = (Theoretical Model)) OR ALL = (Theoretical Models)) OR ALL = (Models, Theoretic)) OR ALL = (Models (Theoretical))) OR ALL = (Experimental Model)) OR ALL = (Model, Experimental)) OR ALL = (Models, Experimental)) OR ALL = (Experimental Models))
2. ((((((((((((((((((((((ALL = (Mathematical Model)) OR ALL = (Mathematical Models)) OR ALL = (Model, Mathematical)) OR ALL = (Models, Mathematical)) OR ALL = (Theoretical Study)) OR ALL = (Studies, Theoretical)) OR ALL = (Study, Theoretical)) OR ALL = (Theoretical Studies)) OR ALL = (Regression Analysis)) OR ALL = (Regression Analysis)) OR ALL = (Analysis, Regression)) OR ALL = (Analyses, Regression)) OR ALL = (Regression Analyses)) OR ALL = (Regression Diagnostics)) OR ALL = (Diagnostics, Regression)) OR ALL = (StatisticalRegression)) OR ALL = (Regression, Statistical)) OR ALL = (Regressions, Statistical)) OR ALL = (Statistical Regression)) OR ALL = (Biopsychosocial Models)) OR ALL = (Model, Biopsychosocial)) OR ALL = (Biopsychosocial Model)
3. (((((((((((((((((((((((ALL = (Body Images)) OR ALL = (Image, Body)) OR ALL = (Body Identity)) OR ALL = (Identity, Body)) OR ALL = (Body Representation)) OR ALL = (Body Representations)) OR ALL = (Representation, Body)) OR ALL = (Body Schema)) OR ALL = (Body Schemas)) OR ALL = (Schema, Body)) OR ALL = (Dissatisfaction, Body)) OR ALL = (Body Image Dissatisfaction)) OR ALL = (Body Image dissatisfaction)) OR ALL = (Dissatisfaction, Body Image)) OR ALL = (dissatisfaction, Body Image)) OR ALL = (ImageDissatisfaction, Body)) OR ALL = (Image dissatisfaction, Body)) OR ALL = (Negative Body Image)) OR ALL = (Body Image, Negative)) OR ALL = (Body Images, Negative)) OR ALL = (Image, Negative Body)) OR ALL = (Images, Negative Body)) OR ALL = (Negative Body Image)) OR ALL = (body image)

Search strategy—EMBASE

((‘postpartum’/exp OR postpartum) AND period OR (period, AND (‘postpartum’/exp OR postpartum)) OR ‘postpartum’/exp OR postpartum OR ((‘postpartum’/exp OR postpartum) AND (‘women’/expOR women)) OR ((‘women,’/exp OR women,) AND (‘postpartum’/exp OR postpartum)) OR ‘puerperium’/exp OR puerperium) AND (latent AND class AND (‘analysis’/exp OR analysis) OR (latent AND class AND analyses) OR (latent AND variable AND (‘modeling’/exp OR modeling)) OR (latent AND variable AND modelings) OR ((‘modeling,’/exp OR modeling,) AND latent AND variable) OR (variable AND (‘modeling,’/exp OR modeling,) AND latent) OR (structural AND (‘equation’/expOR equation) AND (‘modeling’/exp OR modeling)) OR ((‘modeling,’/exp OR modeling,) AND structural AND (‘equation’/exp OR equation)) OR (structural AND (‘equation’/exp OR equation) AND modelings) OR (probabilistic AND latent AND semantic AND (‘analysis’/exp OR analysis)) OR (latent AND class AND (‘model’/exp OR model)) OR (latent AND class AND (‘models’/exp OR models)) OR ((‘model,’/exp OR model,) AND latent AND class) OR (models, AND theoretical) OR ((‘model,’/expOR model,) AND theoretical) OR (theoretical AND (‘model’/exp OR model)) OR (theoretical AND (‘models’/exp OR models)) OR (models, AND theoretic) OR ((‘models’/exp OR models) AND theoretical) OR (experimental AND (‘model’/exp OR model)) OR ((‘model,’/exp OR model,) AND experimental) OR (models, AND experimental) OR (experimental AND (‘models’/exp OR models)) OR (mathematical AND (‘model’/exp OR model)) OR (mathematical AND (‘models’/exp OR models)) OR ((‘model,’/exp OR model,) AND mathematical) OR (models, AND mathematical) OR (theoretical AND (‘study’/exp OR study)) OR (studies, AND theoretical) OR ((‘study,’/exp OR study,) AND theoretical) OR (theoretical AND (‘studies’/exp OR studies)) OR ((‘regression’/exp OR regression) AND (‘analysis’/expOR analysis)) OR ((‘analysis,’/exp OR analysis,) AND (‘regression’/exp OR regression)) OR (analyses, AND (‘regression’/exp OR regression)) OR ((‘regression’/exp OR regression) AND analyses) OR ((‘regression’/exp OR regression) AND (‘diagnostics’/exp OR diagnostics)) OR ((‘diagnostics,’/expOR diagnostics,) AND (‘regression’/exp OR regression)) OR (regression, AND statistical) OR (regressions, AND statistical) OR (statistical AND (‘regression’/exp OR regression)) OR (biopsychosocial AND (‘models’/exp OR models)) OR ((‘model,’/exp OR model,) AND biopsychosocial) OR (biopsychosocial AND (‘model’/exp OR model))) AND ((‘body’/exp OR body) AND images OR (image, AND (‘body’/exp OR body)) OR ((‘body’/exp OR body) AND (‘identity’/exp OR identity)) OR ((‘identity,’/exp OR identity,) AND (‘body’/exp OR body)) OR ((‘body’/exp OR body) AND representation) OR ((‘body’/exp OR body) AND representations) OR (representation, AND (‘body’/exp OR body)) OR ((‘body’/exp OR body) AND schema) OR ((‘body’/exp OR body) AND schemas) OR (schema, AND (‘body’/exp OR body)) OR (dissatisfaction, AND (‘body’/expOR body)) OR ((‘body’/exp OR body) AND (‘image’/exp OR image) AND (‘dissatisfaction’/expOR dissatisfaction)) OR ((‘body’/exp OR body) AND (‘image’/exp OR image) AND dissatisfactions) OR (dissatisfaction, AND (‘body’/exp OR body) AND (‘image’/exp OR image)) OR (dissatisfactions, AND (‘body’/exp OR body) AND (‘image’/exp OR image)) OR ((‘image’/exp OR image) AND dissatisfaction, AND (‘body’/exp OR body)) OR ((‘image’/exp OR image) AND dissatisfactions, AND (‘body’/exp OR body)) OR ((‘body’/exp OR body) AND image, AND negative) OR ((‘body’/exp OR body) AND images, AND negative) OR (image, AND negative AND (‘body’/exp OR body)) OR (images, AND negative AND (‘body’/expOR body)) OR (negative AND (‘body’/exp OR body) AND (‘image’/exp OR image)) OR ((‘body’/expOR body) AND (‘image’/exp OR image)))

Search strategy—American Psychological Association

(((((((Postpartum Period) OR (Period, Postpartum)) OR (Postpartum)) OR (Postpartum Women)) OR (Women, Postpartum)) OR (Puerperium)) AND (((((((((((((((((((((((((((((((((((((((((((((((Latent ClassAnalysis) OR (Latent Class Analysis)) OR (Latent Class Analyses)) OR (Latent Variable Modeling)) OR (Latent Variable Modelings)) OR (Modeling, Latent Variable)) OR (Variable Modeling, Latent)) OR (Structural Equation Modeling)) OR (Modeling, Structural Equation)) OR (Structural EquationModelings)) OR (Probabilistic Latent Semantic Analysis)) OR (Latent Class Model)) OR (Latent ClassModels)) OR (Model, Latent Class)) OR (Models, Theoretical)) OR (Models, Theoretical)) OR (Model, Theoretical)) OR (Theoretical Model)) OR (Theoretical Models)) OR (Models, Theoretic)) OR (Models (Theoretical))) OR (Experimental Model)) OR (Model, Experimental)) OR (Models, Experimental)) OR (Experimental Models)) OR (Mathematical Model)) OR (Mathematical Models)) OR (Model, Mathematical)) OR (Models, Mathematical)) OR (Theoretical Study)) OR (Studies, Theoretical)) OR (Study, Theoretical)) OR (Theoretical Studies)) OR (Regression Analysis)) OR (Regression Analysis)) OR (Analysis, Regression)) OR (Analyses, Regression)) OR (Regression Analyses)) OR (RegressionDiagnostics)) OR (Diagnostics, Regression)) OR (Statistical Regression)) OR (Regression, Statistical)) OR (Regressions, Statistical)) OR (Statistical Regression)) OR (Biopsychosocial Models)) OR (Model, Biopsychosocial)) OR (Biopsychosocial Model))) AND ((((((((((((((((((((((((Body Images) OR (Image, Body)) OR (Body Identity)) OR (Identity, Body)) OR (Body Representation)) OR (Body Representations)) OR (Representation, Body)) OR (Body Schema)) OR (Body Schemas)) OR (Schema, Body)) OR (Dissatisfaction, Body)) OR (Body Image Dissatisfaction)) OR (Body ImageDissatisfactions)) OR (Dissatisfaction, Body Image)) OR (Dissatisfactions, Body Image)) OR (ImageDissatisfaction, Body)) OR (Image Dissatisfactions, Body)) OR (Negative Body Image)) OR (Body Image, Negative)) OR (Body Images, Negative)) OR (Image, Negative Body)) OR (Images, Negative Body)) OR (Negative Body Image)) OR (body image))
